# Autodisplay of Human Hyaluronidase Hyal-1 on *Escherichia coli* and Identification of Plant-Derived Enzyme Inhibitors

**DOI:** 10.3390/molecules200915449

**Published:** 2015-08-26

**Authors:** Zoya Orlando, Isabelle Lengers, Matthias F. Melzig, Armin Buschauer, Andreas Hensel, Joachim Jose

**Affiliations:** 1 Institute of Pharmaceutical and Medicinal Chemistry, Phytochemistry, PharmaCampus, Westfälische Wilhelms-Universität Münster, Corrensstr. 48, 48149 Münster, Germany; E-Mails: zoya.orlando@uni-muenster.de (Z.O.); isabelle.lengers@uni-muenster.de (I.L.); 2 Institute of Pharmacy, Pharmaceutical Biology, Freie Universität Berlin, Königin-Luise Str. 2+4, 14195 Berlin, Germany; E-Mail: melzig@zedat.fu-berlin.de; 3 Department of Pharmaceutical/Medicinal Chemistry II, Institute of Pharmacy, University Regensburg, Universitätsstr. 31, 93040 Regensburg, Germany; E-Mail: armin.buschauer@ur.de; 4 Institute of Pharmaceutical Biology and Phytochemistry, PharmaCampus, Westfälische Wilhelms-Universität Münster, Corrensstr. 48, 48149 Münster, Germany; E-Mail: ahensel@uni-muenster.de

**Keywords:** Autodisplay, Hyal-1, hyaluronan, natural inhibitors

## Abstract

Hyaluronan (HA) is the main component of the extracellular matrix (ECM). Depending on its chain size, it is generally accepted to exert diverse effects. High molecular weight HA is anti-angiogenic, immunosuppressive and anti-inflammatory, while lower fragments are angiogenic and inflammatory. Human hyaluronidase Hyal-1 (Hyal-1) is one of the main enzymes in the metabolism of HA. This makes Hyal-1 an interesting target. Not only for functional and mechanistic studies, but also for drug development. In this work, Hyal-1 was expressed on the surface of *E. coli*, by applying Autodisplay, to overcome formation of inactive “inclusion bodies”. With the cells displaying Hyal-1 an activity assay was performed using “stains-all” dye. Subsequently, the inhibitory effects of four saponins and 14 plant extracts on the activity of surface displayed Hyal-1 were evaluated. The determined IC_50_ values were 177 µM for glycyrrhizic acid, 108 µM for gypsophila saponin 2, 371 µM for SA1657 and 296 µM for SA1641. *Malvae sylvestris* flos, *Equiseti herba* and *Ononidis radix* extracts showed IC_50_ values between 1.4 and 1.7 mg/mL. In summary, Autodisplay enabled the expression of functional human target protein Hyal-1 in *E. coli* and facilitated an accelerated testing of potential inhibitors.

## 1. Introduction

### Hyaluronan (HA) and Human Hyaluronidase Hyal-1 (Hyal-1)

HA is a negatively charged linear biopolymer, which consists of d-glycuronic acid and *N*-acetylglucosamine disaccharide units. Usually, the polysaccharide contains 2000–30,000 disaccharide units, reaching a molecular mass up to 10^7^ Da. HA is the main component of the extracellular matrix and is prominent in skin, vitreous of eyes, synovial fluid and periodontal connective soft tissue. In contrast to other glycosaminoglycans, it is not sulphated and is produced by hyaluronan synthases on the inner side of the plasma membrane. HA has diverse biological activities. It is involved in processes like cell proliferation, migration and cell differentiation [1]. The interaction of HA with cell surface receptors like CD44 is associated with mediation of intracellular signals and behaviour such as matrix-cell signalling and cell-cell aggregation [2]. The biological and pathophysiological roles of HA largely depend on its chain size. High molecular mass HA, with a molecular weight about >20 kDa, has anti‑inflammatory, immunosuppressive, anti-angiogenic and space filling effects. In contrast, low molecular mass HA (<20 kDa) stimulates inflammation, invasiveness and angiogenesis by enhancing endothelial cell migration [3,4,5]. Expression of both IL-1β and TNFα can be induced by low molecular weight HA and it is able to activate the NF-κB/I-κBα autoregulatory mechanism in macrophages [6]. The degradation of high molecular weight HA into smaller fragments is caused by hyaluronidases. Six human hyaluronidases are known. Mainly Hyal-1, Hyal-2 and PH-20 are crucial for regulation of steady-state levels of HA deposit [7]. The human hyaluronidases, Hyal-3, Hyal-4 and pHYAL1, appear to contribute no effect in HA metabolism [8]. Human Hyal-1 is the major hyaluronidase in somatic tissue and was found at high levels in major organs such as liver, kidney, heart and spleen. It is a lysosomal enzyme with three *N*-glycosylation sites, and degrades high molecular mass HA into smaller fragments, in particular tetrasaccharides [9]. It has been shown that Hyal-1 expression is elevated in a variety of cancer cells, for example in prostate, bladder and head tumour cells [10,11]. Furthermore a correlation between Hyal-1 overexpression and the malignancy of human cancer cell lines MCF-7 and MDA-MB-231 has been reported [12]. Because of its role in these (patho)physiological processes, Hyal-1 is an interesting target for drug development and inhibitor testing. The inhibition of Hyal-1 using hyaluronidase inhibitors might be a new additive way in more targeted cancer treatment or in treatment of non‑cancer diseases such as arthritis and gingivitis [13,14]. Potent and specific inhibitors of Hyal-1 are not known, so far. Only a few small molecules as inhibitors towards purified recombinant Hyal-1 are identified such as glycerrhizic acid (IC_50_ = 39.4 µM) as naturally occurring compound [15] or l-ascorbic acid tridecanoate (IC_50_ = 50 ± 4 µM) [16]. In summary, the number of known Hyal-1 inhibitors is very limited. This fact and an increasing knowledge of pathophysiological roles of hyaluronidases make the search for new inhibitors necessary. In the present study, autodisplayed Hyal-1 on the surface of *E. coli* was used to test potential inhibitors. Autodisplay takes advantage of the natural autotransporter secretion mechanism of Gram-negative bacteria, e.g., *E. coli* [17]. It was reported to enable the cell surface display of a wide variety of recombinant proteins and peptides, so-called passengers. There were several successfully expressed enzymes using Autodisplay, among them human CK2 [18], sorbitol dehydrogenase [19], nitrilases [20,21], β-lactamase [22], human hyaluronidase PH-20 [23], cytochromes P450 [24,25], esterases [26,27,28] and lipase [29]. Besides displaying enzymes, Autodisplay can also be utilized for the surface display of enzyme inhibitors, epitopes and peptide libraries [30]. Still, the most convenient feature of Autodisplay, using well‑studied bacterium *E. coli* as a rapid production system, is its cost and time efficiency. The application of whole cells displaying enzymes prevents the time consuming and costly process of cell disruption and enzyme purification. In case of human hyaluronidase Hyal-1, an expression of functional enzyme was to date only possible in eukaryotic systems. Expression of this enzyme in *E. coli* yielded inactive inclusion bodies and made refolding steps necessary in order to obtain the enzyme in an active form [16,31]. By applying Autodisplay, the formation of inclusion bodies is avoided due to the immediate translocation of the fusion protein across the bacterial cell membranes. This resulted in functional surface displayed Hyal-1 on the surface of *E. coli* and facilitated screening of potential inhibitors.

## 2. Results and Discussion

### 2.1. Artificial Gene Construction for the Surface Display of Hyal-1

**Figure 1:**
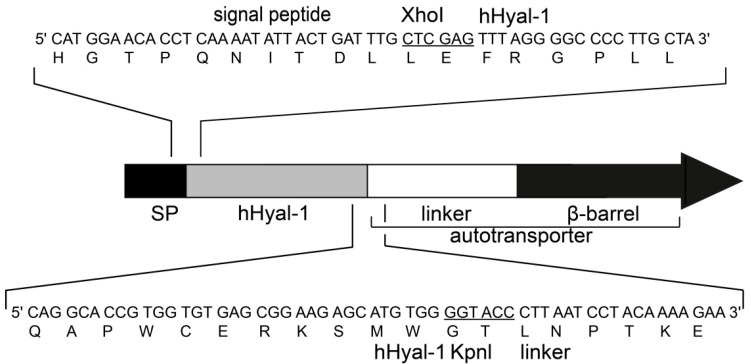
Schematic structure of the Hyal-1 autotransporter fusion protein. The DNA- and amino acid sequences are plotted. Restrictions endonucleases recognition sites for Xhol and KpnI, used for the construction of the artificial gene encoding the fusion protein, are underlined.

The gene of Hyal-1 was amplified and fused at the 5′end to the gene encoding the signal peptide of choleratoxin-B (CtxB) and at the 3′end to the gene encoding the adhesion involved in diffuse adherence-I (AIDA-I) transport unit. Thereby, the polymerase chain reaction (PCR) product of Hyal-1 DNA-sequence without the coding sequence of the eukaryotic signal peptide was cleaved with enzymes XhoI and KpnI [9]. The plasmid pJM007, containing all required domains for surface display of CtxB, was used as the acceptor vector [32]. Cleavage of this plasmid by XhoI/KpnI resulted in a deletion of the DNA-sequence of the original passenger CtxB (Figure 1). Ligation of cleaved PCR product and plasmid pJM007 resulted in the plasmid pAK009, which directs the expression of the fusion protein under control of the constitutive P_TK_ promotor [32]. Due to the ligation procedure the produced fusion protein consists of the CtxB signal peptide, Hyal-1 as passenger, the linker region and the β-barrel (Figure 1). The *E. coli* F470 strain was transformed with the resulting plasmid pAK009. *E. coli* F470 is lacking the *O*-polysaccharide of the lipopolysaccharide (LPS) to avoid inhibition of Hyal-1 by the sugar moieties of intact LPS [23,33]. The predicted molecular mass of the fusion protein after processing by the signal peptidase was approximately 98 kDa.

### 2.2. Surface Display of Hyal-10

#### 2.2.1. Whole Cell Enzyme Linked Immunosorbent Assay (ELISA)

**Figure 2:**
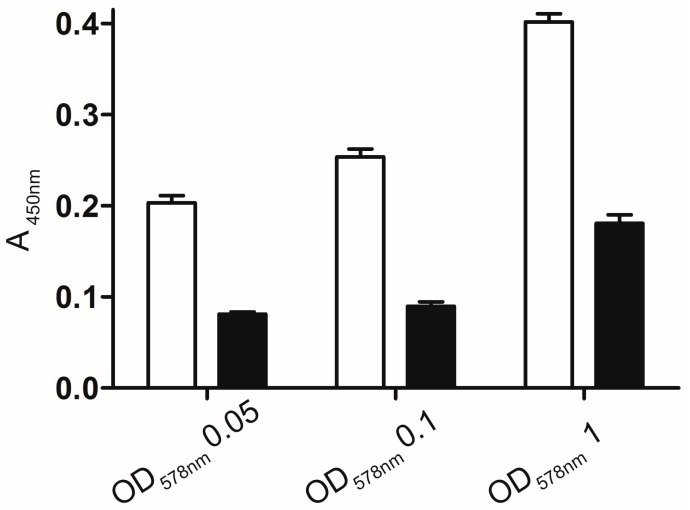
Whole cell enzyme-linked immunosorbent assay (ELISA). White: *E. coli* F470 cells containing pAK009 for surface displaying of Hyal-1. Black: *E. coli* F470 cells without plasmid. Wells of a Maxisorp^®^ 96-plate were coated with cell suspensions of various optical densities at 578 nm (OD_578_) of 0.05; 0.1 and 1. After labelling with the primary anti-Hyal-1 antibody and incubation with a secondary antibody conjugated with horse radish peroxidase the reaction was started by adding of 3,3′,5,5′-tetramethylbenzidine (TMB). A light‑protected incubation was followed for 10 min at RT. Subsequently, the reaction was stopped by adding sulphuric acid. The absorbance was recorded at 450 nm (*n* = 3, error bars ± SD).

The wells of a Maxisorp^®^ 96-well-platte were coated with *E. coli* F470 pAK009 cells. *E. coli* F470 cells carrying pJM007 and displaying CtxB, the β-subunit of cholera toxin, were applied as a control to identify a possible false positive cross reaction with other parts of the fusion protein [32]. First, the wells were coated with *E. coli* F470 cells carrying the corresponding plasmids. After removing the unbound cells, the wells were blocked with 5% milk powder suspension. Before a primary polyclonal murine anti-Hyal-1 antiserum was added and incubated, the wells were washed three times with PBS-Tween 20. A secondary horse radish peroxidase (HRP)—coupled antibody was added, after removing the primary anti-Hyal-1 antiserum by three repeated washing steps with PBS‑Tween 20. The secondary anti mouse antibody was removed and the wells were washed again as well as performed before. Thereafter, the detection reagent, 3,3′,5,5′-tetramethylbenzidine (TMB), was added to each well. Application of sulphuric acid resulted in the formation of a yellow colour detectable at 450 nm. A significant stronger, dose dependent colour formation was detected with cells displaying Hyal-1 when compared to the colour formation of control cells (Figure 2). This was a strong hint for a surface display of Hyal-1 by *E. coli* F470 carrying pAK009.

#### 2.2.2. Protease Accessibility Test

**Figure 3:**
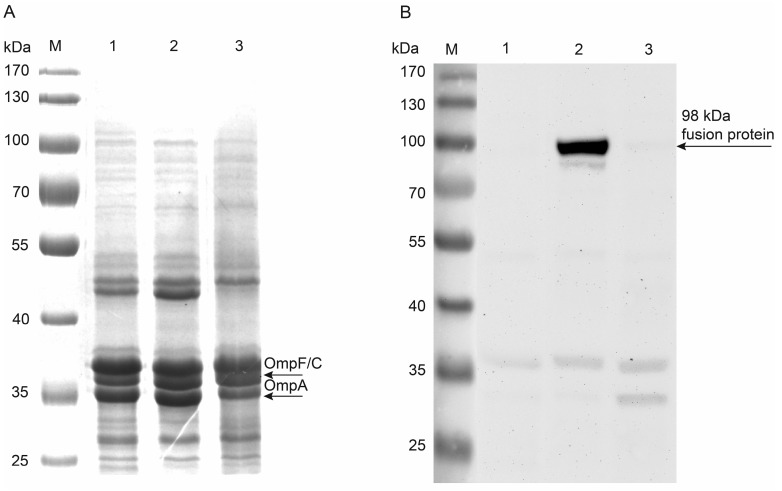
Surface exposure of Hyal-1 on *E. coli* F470. M: PageRuler Prestained Protein Ladder (Fermentas); Lane 1: *E. coli* F470 without plasmid (control sample); Lane 2: *E. coli* F470 + pAK009; Lane 3: *E. coli* F470 + pAK009 with whole cell digestion using proteinase K before membrane protein preparation. (**A**) Coomassie stained SDS‑PAGE of enriched outer membrane protein from *E. coli* F470. Samples were dissolved in denaturation buffer containing 2% SDS and 100 mM dithiothreitol (DTT) (final concentration) and heated for 10 min at 95 °C; (**B**) Antigenic evaluation of enriched outer membrane proteins from *E. coli* F470 after SDS-PAGE with 10% PA and western blot. For detection, polyclonal murine anti-Hyal-1 antibody and the secondary anti‑mouse IgG antibody conjugated with horse radish peroxidase (HRP) were used. Visualisation was performed using ECL reagent. Chemoluminescence was detected after an exposure time of 30 min and 30 s, documented in inverse mode.

In order to examine further, whether Hyal-1 was expressed at the cell surface of *E. coli* F470 pAK009, a protease accessibility test was performed. A protease, such as proteinase K, is not able to cross the membrane barrier and hence can only digest proteins, which are accessible from the extracellular side. A digestion by externally added proteinase K strongly indicates the surface display of a protein [34]. After treating *E. coli* F470 cells without plasmid, cells with plasmid pAK009, and cells with plasmid pAK009 with proteinase K, a preparation of the outer membrane proteins was performed. The samples were separated via SDS-PAGE with 10% PA and stained with Coomassie Brilliant Blue. As shown in Figure 3A, Coomassie staining was not sensitive enough to detect a protein in the range of 98 kDa as expected for Hyal-1 fusion protein. This could have been due a relatively low amount of expressed fusion protein compared to the total amount of outer membrane proteins. Therefore, immunoblot analysis of the same protein preparations was performed. The western blot was treated with a specific polyclonal murine antiserum against Hyal-1. After washing the membrane with TBS-Tween 20, it was incubated with a secondary anti mouse antibody (HRP-coupled). Subsequently, the membrane was washed a second time, and the luminescence reagents luminol A and B (ECL reagent) were added. As expected, chemiluminescence detection revealed the presence of the fusion protein with a correct size of 98 kDa, in the sample of cells carrying pAK009 not treated with proteinase K and was only weakly present in the sample treated with proteinase K (Figure 3B) as well as in the control cells not carrying pAK009. An abundant outer membrane protein is OmpA (*ca.* 35 kDa), which is visible by Coomassie staining after SDS-PAGE. OmpA comprises a C-terminal protease sensitive extension protruding in the periplasm of *E. coli*. A reduction in the molecular weight of OmpA from 35 kDa to molecular weight lower than 25 kDa would have indicated that the periplasm was not protected from proteinase K and the integrity of the outer membrane was disturbed during digestion. Since a band of OmpA with an approximately size of 35 kDa in sample of proteinase K treated cells was clearly detectable at similar amounts as in cells not treated with protease (Figure 3A, lane 3), the outer membrane must have been intact and proteinase K only digested the accessible Hyal-1 on the cell surface. In summary, surface display of Hya1-1 on *E. coli* F470 could be verified by the protease accessibility test.

In order to improve the amount of Hyal-1 on the surface of *E. coli*, we expressed the same Hyal-1 construct under the control of a T7/lac promotor. After induction of protein expression with 1 mM IPTG, a significantly increased amount of Hyal-1 was present in outer membrane preparation, an amount that was easily detectable by SDS-PAGE with 10% PA. However, these cells exhibited almost no enzymatic activity (data not shown). This could have been a hint that the rapid expression by the inducible promoter led to a large portion of misfolded enzyme. Therefore we decided to continue to work with the Hyal-1 construct under control of the constitutive promoter.

### 2.3. Hyaluronidase Activity of Whole Cells Displaying Hyal-1

#### 2.3.1. Enzyme Activity Determination and pH Dependency

**Figure 4:**
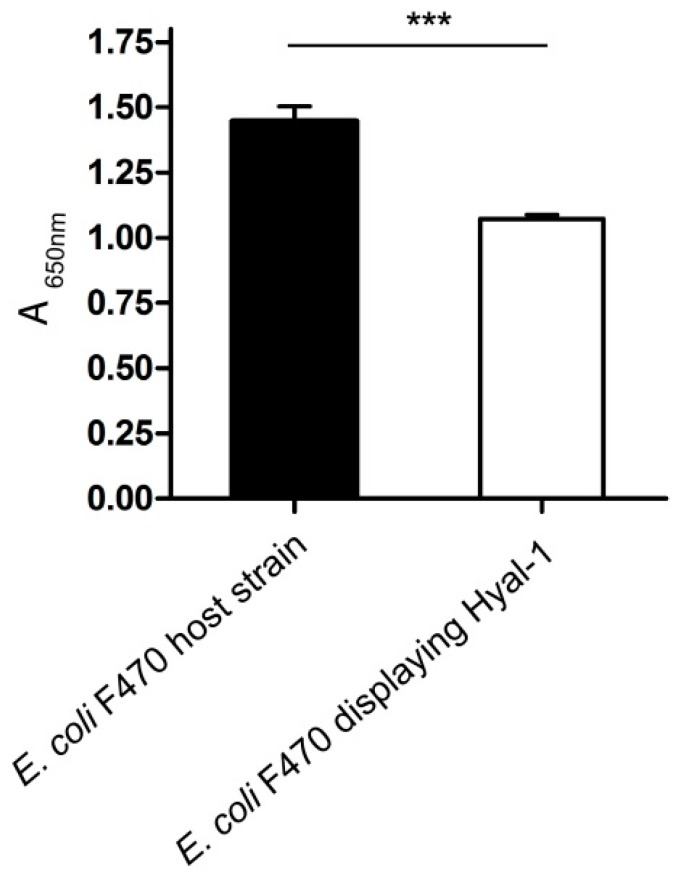
Activity determination of Hyal-1 displayed on the cell surface of *E. coli* F470. *E. coli* F470 displaying Hyal-1 and the control sample *E. coli* F470 not displaying Hyal-1 (host strain) were suspended in formate buffer (pH 3.5), and mixed with the substrate solution resulting in a final OD_578_ of 10 and HA final concentration of 0.11 mg/mL. After an incubation time of 5 min the cells were removed by a centrifugation step at 4 °C. 25 µL of the supernatant was applied to the wells of a 96-microplate and mixed with “stains-all” solution following deionized water. A decrease in absorbance indicates activity. The absorbance was measured directly at 650 nm. Data are shown as mean values. (*n* = 3, error bars ± SD, *** *p* < 0.05, unpaired *t* test).

Initially activity determination of displayed Hyal-1 on *E. coli* F470 was performed by utilization of formate buffer (pH 3.5, Figure 4). Absorbance at 650 nm was measured in comparison to *E. coli* F470 not carrying pAK009 and therefore not displaying Hyal-1. After harvesting, the cells were suspended in buffer with a final OD_578_ of 20. The enzymatic reaction started by adding the substrate (final OD_578_ 10). The decrease in absorbance measured for *E. coli* F470 Hyal-1 was due to the degradation of high molecular weight HA by the enzyme displayed at the surface of the bacteria cell and indicates activity. Intracellular expression of Hyal-1 in *E. coli* was found to be not functional [31]. By applying the Autodisplay technology the fusion protein is connected to a sec (secretion) signal peptide. Thereby the intracellular chaperones of the sec machinery holds the protein in an unfolded form until transport [35]. The proximate direction of the fusion protein to the secretion machinery by signal peptide could have prevented the formation of intracellular aggregates of the protein. The substrate HA, a polysaccharide, is a large molecule and cannot cross the outer membrane of *E. coli*. Thus, cleavage of the substrate was restricted to the cell surface.

**Figure 5:**
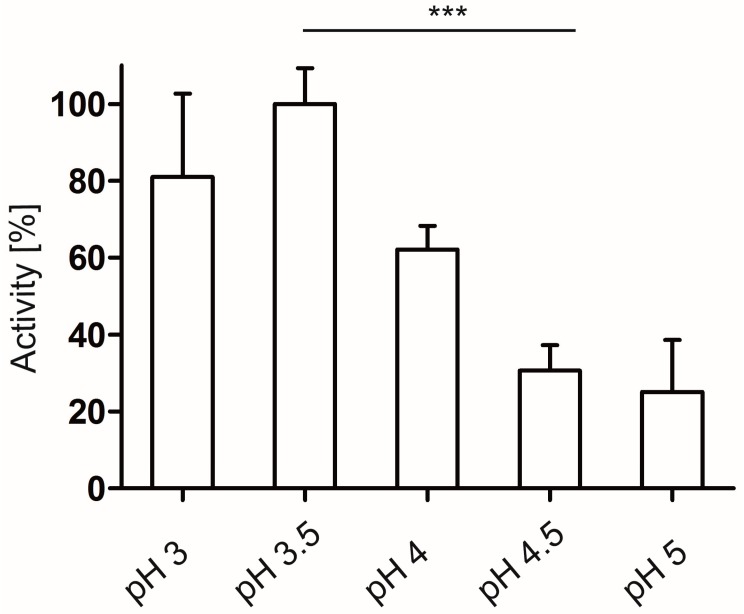
Activity measurement of Hyal-1 displayed on *E. coli* F470 at different pH values. *E. coli* F470 displaying Hyal-1 was suspended in formate buffer, pH ranges 3–5 and mixed with the substrate solution resulting in a final OD_578_ of 10 and HA final concentration of 0.11 mg/mL. After an incubation time of 5 min the cells were removed by a centrifugation step at 4 °C. 25 µL of the supernatant was applied to the wells of a 96-microplate and mixed with “stains-all” solution following deionized water. The absorbance was immediately measured at 650 nm. The highest decrease in absorbance due to the cleavage of HA was set 100% activity. Mean values are given. There were no significant differences in activity at pH 3 and 3.5 as well as in activity at pH 4.5 and 5 (*n* = 3, error bars ± SD, *** *p* < 0.05, unpaired *t* test).

Human Hyal-1 is a lysosomal enzyme and was reported to exhibit a narrow acidic pH optimum (3–4) for the cleavage of HA [36,37]. The influence of *E. coli* cells on the pH dependency of the Hyal-1 activity was investigated. *E. coli* F470 cells carrying pAK009 were cultured as described below. Except of the pH values of the formate buffer, the same approach and reaction conditions as described above were used. The enzymatic reaction was carried out at pH 3; 3.5; 4; 4.5 and 5. Due to the fact that the buffer capacity of formate buffer is not given at pH above 5, the activity determination was not performed at higher pH than 5. In order to keep comparable reaction conditions, beside the pH value, a change in the buffering system was avoided. The highest activity was obtained at pH 3.5, however with no significant difference in activity achieved at pH 3 (Figure 5). Higher pH resulted in a significant loss of activity. This is in good agreement with the reported pH optimum for Hyal-1 activity and consistent with the low pH in the lysosomes. In consequence further investigations on enzyme activity in this study were carried out at pH 3.5.

#### 2.3.2. Optimal Time and Cell Density

**Figure 6:**
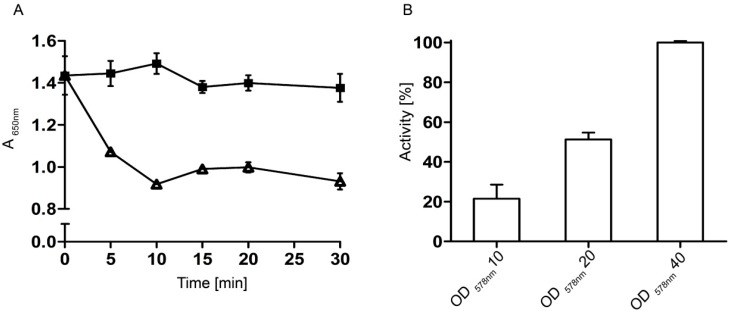
Optimal time (**A**) and cell density (**B**) for measuring the Hyal-1 activity displayed on *E. coli* F470; (**A**) Triangles: *E. coli* F470 pAK009 displaying Hyal-1; black quadrats: control cells without plasmid. The cell suspensions (final OD_578_ of 10) were incubated with HA (0.11 mg/mL) over a period of 10 min. After separating the cells by centrifugation, the supernatant was mixed with stabilised “stains-all” solution and deionized water. The absorbance was measured at 650 nm; (**B**) *E. coli* F470 with pAK009 were resuspended in buffer pH 3.5 and mixed with HA (0.11 mg/mL) to give final optical densities (OD_578_) of 10, 20, and 40. After an incubation time of 5 min at 37 °C, cells were removed and the supernatant was measured as described in (**A**). The highest decrease in absorbance was set 100% activity. The mean values are shown (*n* = 3, error bars ± SD, unpaired *t* test).

Prior to compound screening, the optimal reaction time was determined. The cells were prepared and mixed with HA as described. A final OD_578_ of 10 was set. To ensure that the metabolites of *E. coli* do not interfere with the assay setup, *E. coli* F470 cells without plasmid were prepared as control. In Figure 6A the time dependent curve of absorbance at 650 nm is shown. A significant decrease in absorbance was detected over a period of 10 min in sample with cells displaying Hyal-1. The reaction velocity was found to be nearly linear during these 10 min. Due to this finding an incubation time of 5 min was chosen for all following measurements. To normalize the absorption values obtained with surface displayed Hyal-1, an additional “stains-all” assay with 12.5 U/mL of ovine testis hyaluronidase (OTH) was performed and showed a slope of 20 mAU/min (data not shown). In comparison, the *E. coli* F470 cells displaying Hyal-1 with an OD_578_ of 10 yielded a decrease in absorbance of 363.5 mAU or a slope of 72.7 mAU/min respectively, after 5 min reaction time. This allowed to calculate the enzymatic activity of the cell suspension to correspond to an enzyme activity of 45.44 U OTH. An *E. coli* cell suspension with an OD_578_ of 10 contains about 8.6 × 10^8^ cells/mL [18]. The enzyme activity of a single cell of *E. coli* F470 displaying Hyal-1 was calculated to be about 4.54 × 10^‑9^ U under these conditions. To show a relation between the amount of cells displaying Hyal-1 and hyaluronidase activity, cells were incubated at various optical densities with HA. As expected, hyaluronidase activity increased with cell concentrations displaying Hyal-1 (Figure 6B). For inhibitor screening an OD_578_ of 10 was defined to avoid potential interactions between the screened compounds and a high number of *E. coli* F470 cells. Nevertheless, by applying this optical density of cells the hyaluronidase activity was sufficient for testing inhibitors. In addition a abandoning of the linear curve area after 5 min by higher hyaluronidase activity due to the higher OD of the cells displaying Hyal-1 could be avoided.

### 2.4. Inhibitor Testing with Surface Displayed Hyal-1

**Figure 7:**
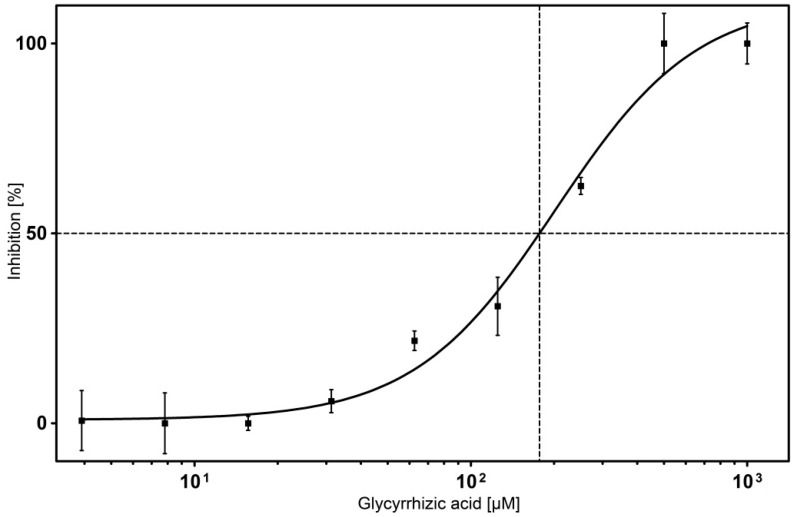
IC_50_ value determination of glycyrrhizic acid. Inhibition of surface displayed Hyal-1 was measured after incubation with glycyrrhizic acid at concentrations ranging from 0 to 1 mM. Calculation of IC_50_ values was done using GraphPadPrism5 Software. The IC_50_ value was calculated from the inflectionpoint (50% inhibition of the Hyal-1 activity) of the plot (*n* = 3, error bars ± SD).

It was challenging to select compounds for inhibitor testing with surface displayed Hyal-1, because low molecular inhibitors of Hyal-1 are rarely known. In order to proof the test system, we decided to choose glycyrrhizic acid as a reference inhibitor. Glycyrrhizic acid is a triterpenoid saponin glycoside, which has been proven to exhibit an inhibition towards Hyal-1 in a micromolar range [15]. The IC_50_ value of glycyrrhizic acid was determined in this study. Therefore a dose-response analysis in concentrations ranging from 0 to 1 mM was performed. 100% activity was set for samples in the presence of dimethylsulfoxide (DMSO) however in the absence of potential inhibitors. A “sigmoid curve with variable slope” was the analysis option for the calculation by GraphPadPrism5 (GraphPad, La Jolla, CA, USA, Figure 7). The concentration of the compound at the inflectionpoint on a semi logarithmic dose-response plot represented the IC_50_ value. It turned out to be 177 µM for glycyrrhizic acid. By means of an ELISA-like assay and applied partially purified Hyal-1 (from the medium of bladder cancer cells) the identified IC_50_ value of glycyrrhizic acid was 39.4 µM. Human umbilical cord HA, which has a molecular weight of maximum 750 kDa was used [15]. Via turbidimetric assay the inhibitory potential of glycyrrhizic acid, with an IC_50_ value of 26 µM towards recombinant hHyal-1 containing a C-terminal His-tag expressed by *Drosophila* Schneider-2 cells was determined by Hofinger *et al.* [16]. Autodisplay of Hyal-1 on the surface of *E. coli* F470 offers no opportunity of posttranslational modifications, such as glycosylation. Human Hyal-1 has three *N*-glycosylation sites located at positions N-99, N-216 and N-350. As reported earlier, deglycosylation of recombinant Hyal-1 expressed by eukaryotic Schneider-2 cells led to a decrease in enzymatic activity of about 40% [16]. The missing glycosylation of Hyal-1 displayed on the surface of *E. coli* may have contributed to a reduced enzyme activity. In general, missing posttranslational modifications could lead to differences in the determined inhibition values obtained with the diverse expression systems.

**Figure 8:**
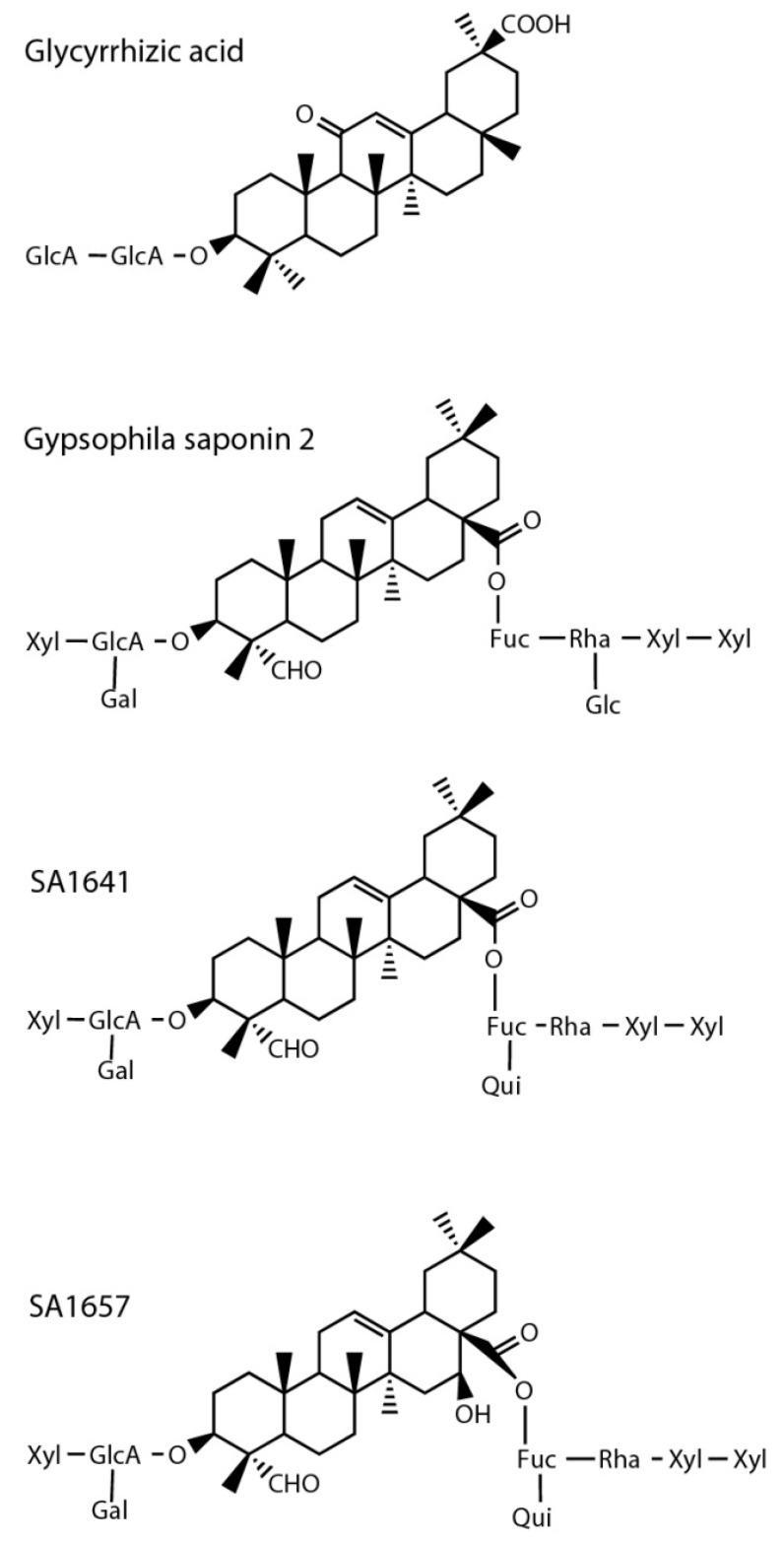
Compounds tested on inhibition of Hyal-1 of displayed on the surface of *E. coli* F470. The abbreviations Fuc (fucose), Gal (galactose), GlcA (glucuronic acid), Glc (glucose), Qui (chinovose), Rha (Rhamnose), Xyl (xylose) stand for different sugar residues.

According to the determination of the IC_50_ value of glycyrrhizic acid, the IC_50_ values of three triterpenoid saponins on Hyal-1 activity were determined as well. As structurally related compounds, gypsophila saponin 2, SA1657 and SA1641 were tested (Figure 8; Table 1) [38,39,40].

Gypsophila saponin 2, SA1657 and SA1641 showed similar IC_50_ values (Table 1). The differences could rely on the deviation in the attached sugar moieties. Interestingly, the only structural difference between SA1641 and SA1657, is the hydroxyl group at the C16. IC_50_ values as determined are in the same order of magnitude as that of the best known inhibitor of Hyal-1 until now, glycyrrhizinic acid, which was determined to be 177 µM in the same assay. Using Hyal-1 prepared from Schneider-2 cells, the IC_50_ value of glycyrrhizinic acid was determined to be 26 µM. As discussed above, this difference by a factor of 6.8 could have been due to the different expression systems, with or without postranslational modification. Nevertheless, triterpenoid saponines appear to be an interesting group of substances for the inhibition of Hyal-1.

**Table 1:** Effect of saponin derivatives on the activity of surface displayed Hyal-1.

Compound	IC_50_ Value [µM]
**Gypsophila saponin 2**	108
**SA1657**	371
**SA1641**	296

Further studies are required to find out, whether the lipophilic triterpenoid/steroidal scaffold, the attached sugars or both of them are involved in the inhibition of Hyal-1. A larger library should be investigated for an evident statement of structure-activity relationship. Flavonoids and saponins, as plant-derived inhibitors of hyaluronidases, were subjects of many studies [41,42,43]. In most studies, the source of enzyme was bovine testis hyaluronidase (BTH), venom hyaluronidases or bacterial lyases. In the present study we analysed a small selection of extracts of traditionally used medicinal plants with no toxic side effects and examined the effect of these plant preparations on Hyal-1 activity (Table 2) [44].

**Table 2:** Effect of plant extracts on the activity of surface displayed Hyal-1.

Plant Extract	Inhibition % [10 mg/mL]	IC_50_ Value [mg/mL]
*Hennae folium*	0	n.d.
*Equiseti herba*	100	1.5
*Betulae folium*	61	n.d.
*Ononidis radix*	81	1.7
*Bucco folium*	21	n.d.
*Maydis stigma*	47	n.d.
*Malvae sylvestris flos*	100	1.4
*Solidaginis herba*	100	4.9
*Chebulae fructus*	0	n.d.
*Coptis rhizome*	0	n.d.
*Cranberry*	10	n.d.
*Althaeae radix*	60	n.d.
*Hydrastis rhizoma*	7	n.d.
*Mahoniae radix*	26	n.d.

n.d.: not determined.

There was no interference detected between the constituents of the plant extracts and the used dye “stains-all”. These extracts predominantly contained either flavonoids, saponins or acidic polysaccharides. In addition, tannins could be included in the preparations. Tannins could act as protein precipitating agents [45]. To examine the influence of tannin agents of the activity of surface displayed Hyal-1, *Chebulae fructus* extract (containing tannins up to 45%) was tested as well. The dried extracts were applied in a final concentration of 10 mg/mL. As shown in Table 2, the extracts of *Equiseti herba*, *Ononidis radix*, *Malveae sylvestris* flos and *Solidaginis herba* showed the most effective inhibition and were subjected for determinations of IC_50_ value.

**Figure 9:**
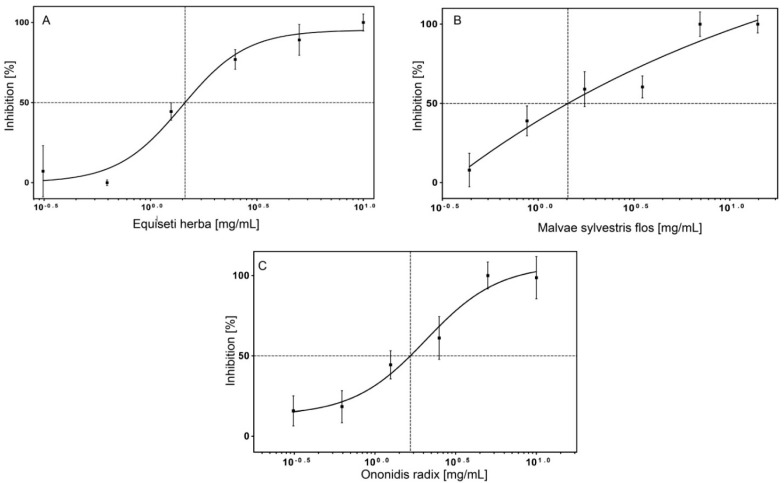
IC_50_ values of different plant extracts on surface displayed Hyal-1. (**A**) *Equiseti herba*; (**B**) *Malvae sylvestris flos*; (**C**) *Ononidis radix*. Activity of surface displayed Hyal-1 was measured after incubation with extract of each plant at concentrations ranging from 0 to 10 mg/mL. The IC_50_ value was calculated from the inflection point (50% inhibition of the Hyal-1 activity) of the plot. The different shape of the curves probably results from the heterogeneous composition of the extracts and from the different solubility of the constituents in the extracts (*n* = 3, error bars ± SD).

The IC_50_ values of extracts of *Malveae sylvestris flos*, *Equiseti herba* and *Ononidis radix* are in a similar range, from 1.4 to 1.7 mg/mL. The measured dose-response curves of these extracts are shown in Figure 9A–C.

The extract of *Solidaginis herba* showed an IC_50_ value of 4.9 mg/mL. These plant extracts contain flavonoids, saponins and polysaccharides in variable amounts. A fractionation of the constituents would help to reveal the active secondary compound from the respective herbal materials or to provide a proof of a synergistically enhanced inhibition by the whole mixture. We suggest, that these extracts could be used as phytotherapeutic preparations in local topical treatment of diseases, in which HA and its metabolism by hyaluronidases are involved. Periodontal diseases belong to such types of sufferings [14]. For example, mouthwashes or gels consisting of these herbal extracts could have a positive impact on the treatment of periodontal disorders. The compounds and plant extracts presented here can be used as starting points for searching inhibitors of Hyal-1 towards the treatment of such disease.

## 3. Experimental Section

### 3.1. Harvesting Whole Cells of E. coli

*E. coli* F470 cells were grown in Luria Bertani (LB) medium for 20 h. Subsequently, the cells were pelleted at 2100× *g* for 2 min at RT and the supernatant was removed. The cells were washed two times in sodium formate buffer (100 mM sodium formate, 100 mM NaCl, pH adjusted by formic acid). For further steps of procedure the cells were resuspended in calculated volume of sodium formate buffer resulting in desired OD_578_.

### 3.2. “Stains-All” Assay

“Stains-all” is a cationic molecule, which forms a blue complex with undegraded HA in presence of water. The product can be quantified at 650 nm. After digestion by hyaluronidases a decrease in absorbance is detectable. The assay procedure for activity measurements of human hyaluronidase PH-20 has been published recently [23]. The compounds and plant extracts were solved in DMSO/ddH_2_O (50:50 (*v*/*v*)). The *E. coli* F470 pAK009 cells (OD_578_ of 20) were preincubated with DMSO or inhibitors with a concentration of interest. For the reaction cell suspensions were mixed with an equal volume of HA (0.22 mg/mL) resulting in a final OD_578_ of 10 and HA final concentration of 0.11 mg/mL. The final concentration of 1% DMSO was tolerated by *E. coli* F470 cells. After incubation at 37 °C for various times (endpoint testing up to 5 min) the enzymatic reaction was stopped by centrifugation of the cells at 4 °C. 25 µL of the supernatant was replaced in a 96‑well plate and mixed with 112 µL of “stains-all” solution (11.2 mg “stains-all”, 17.6 mg ascorbic acid, 13 µL glacial acetic acid, 1 mg butylhydroxytoluol, 50 mL stabilised dioxane, 50 mL ddH_2_O) and with 80 µL water. This was mixed shortly and the absorbance (Awas measured immediately (plate reader Tecan infiniteF200PRO, Männedorf, Swiss) by 10 flashes at the centre of the wells. For calculating of activity, a sample with DMSO served as a reference for 100% activity (DMSO). A control value for no enzyme activity was achieved by applying cells without surface displayed Hyal-1, but containing all other components (control):

(1) $$relative\;activity\;\left[\%\right]=\frac{A\left(control\right)-A\left(inhibitor\right)}{A\left(control\right)-A\left(DMSO\right)}\times 100\%$$

(2) inhibition [%]=100−relative activity [%]

For calculation of the IC_50_ value, several concentrations of every compound or plant extract were analysed in their inhibitory potential. A non-linear curve fitting was used for the analysis. The IC_50_ value was then identified from the midpoint (50% inhibition of Hyal-1 activity) of the plot.

### 3.3. Bacterial Strains, Plasmids and Culture Conditions

*E. coli* strain F470 (F^−^, met^−^, his^−^, pro^−^, mtl^−^, (Str^r^)) was used for the protein expression and was kindly provided by J. Seydel (Center for Medicine and Biosciences, Biophysics, Borstel Research Center, Germany). This strain has a spontaneous mutation in the *rfb*-locus resulting in a reduced lipopolysaccharide (LPS)-layer and therefore this mutation is not part of the annotated genotype of the strain [33,46]. The plasmid pCMVSport6H (ImaGenes gencode: IRATp970C1157D) encoding Hyal-1 was purchased from ImaGenes GmbH (Berlin, Germany). The sequence encoding for Hyal-1 was not codon optimized for *E. coli*. The plasmid pJM007 containing all DNA-sequences of autotransporter domains required for surface displaying and the DNA-sequence of CtxB as passenger was used as backbone for inserting of PCR product of Hyal-1 [32]. The expression via this plasmid is guided by the constitutive inducible promoter P_TK_. *E coli* F470 pAK009 cells were cultivated in 100 mL LB supplemented with 50 µg/mL carbenicillin, 10 µM ethylendiaminetetraacetic acid (EDTA) at 37 °C and 200 rpm. The required LB agar plate contained 50 µg/mL carbenicillin.

### 3.4. Cloning of Artificial Autotransporter Gene Used for Autodisplay

For cloning of Hyal-1 DNA-sequence into the Autodisplay vector the corresponding gene was amplified by PCR using plasmid pCMVSport6H. The oligonucleotides AK001 (5′-CCGCTCGAGTTTAGGGGCCCCTTGCTACC-3′, forward, *Xho*I restriction site underlined) and AK002 (5′-CGGGGTACCCCACATGCTCTTCCGCTC-3′, reverse, *Kpn*I restriction site underlined) were used for the amplification. The PCR program was as follows: initial denaturation step at 94 °C for 5 min, a total of 30 cycles were performed with a denaturation step at 94 °C for 30 s, an annealing step at 65.9 °C for 30 s and an elongation step at 72 °C for 30 s. The final elongation step was followed at 72 °C for 6 min. The PCR product and a DNA preparation of pJM007 were digested with the restriction enzymes XhoI and KpnI. After separation by agarose gel electrophoresis and gel extraction the digested PCR fragment and XhoI/KpnI digested fragment of pJM007 were ligated, resulting in pAK009.

### 3.5. Whole Cell ELISA

The performance of a whole cell ELISA has been described previously [47] and was modified slightly in this work. The *E. coli* F470 pAK009 and the host cells were grown for 20 h in LB medium. After washing in PBS, the cells were suspended to the final OD_578nm_ of 0.05; 0.1 and 1. Subsequently, 100 µL of each resulted suspension was applied to the wells of 96‑well microplate (Maxisorp^®^, Nunc, Langenselbold, Germany). After coating overnight at 4 °C the non‑bounded cells were removed and a blocking step with 150 µL of 5% milk powder in PBS was followed for 3 h, at RT. The blocking buffer was removed and the wells were washed three times with PBS-Tween 20 (0.1% *v*/*v*). 100 µL of a polyclonal murine anti-Hyal-1 antiserum (Abnova, Taipei, Taiwan) diluted 1:1.600 in PBS was added to each well for 1 h, at RT. The wells were rinsed with PBS-Tween 20 and reloaded with an HRP-conjugated anti-mouse IgG diluted 1:10.000 in PBS for 1 h, at RT. After three washing steps, 100 µL of TMB was added to each well for 20 min at RT under light protection. The reaction was stopped by adding of 100 µL 2 M H_2_SO_4_. The absorbance was measured at 450 nm in a plate reader (Tecan infinite F200PRO).

### 3.6. Membrane Protein Isolation

A 100 mL culture of *E. coli* F470 pAK009 was grown for 20 h in LB medium containing 50 µg/mL carbenicillin and 10 µM EDTA at 37 °C and 200 rpm. A culture of *E. coli* F470 without plasmid was prepared, as well. After harvesting cells and washing with 0.2 M Tris/HCl pH 8 membrane proteins were isolated according to a modified protocol of Park *et al.* [48]. The cells were suspended in 5 mL 0.2 M Tris/HCl pH 8. The peptidoglycan layer was hydrolysed by the addition of lysozyme (0.5 mg/mL end concentration) in the presence of 20 mM saccharose and 200 µM EDTA. After 10 min a mixture of aprotinin (0.025 mg/mL), phenylmethylsulfonyl fluoride (PMSF) (1.25 mM) and DNAse (80.75 U/mL) was added, as well as 5 mL extraction buffer (2% Triton-X-100, 50 mM Tris/HCl, 10 mM MgCl_2_). The suspension was kept for 30 min on ice and then centrifuged at 3120× *g* for 5 min. Subsequently, the supernatant was centrifuged at 38,700× *g* for 10 min. The pelleted enriched outer membrane proteins were washed with 10 mL water. An additional centrifugation step followed and the pellet was resuspended in 30 µL water. For the SDS‑PAGE the samples were mixed with an adequate volume (1:1) of protein sample buffer (4% SDS and 20% glycerol) and cooked for 10 min at 95 °C. A proteinase K accessibility test was performed to confirm the surface displaying of Hyal-1. For this purpose *E. coli* F470 pAK009 cells were harvested, washed and suspended in 1 mL buffer (Tris/HCl pH 8). Proteinase K was added at a final activity of 6.1 mAnsonU/mL. This cell suspension was incubated for 60 min at 37 °C. To remove the proteinase K the cells were washed twice with Tris/HCl pH 8 and the membrane proteins were isolated according to the same protocol as described above. Subsequently, the enriched outer membrane proteins were analysed by immunoblot.

### 3.7. Western Blot

The membrane proteins were separated via SDS-PAGE with 10% PA before western blot and antigenic detection were performed. The protein preparations were diluted 1:1 with reducing buffer (100 mM Tris/HCl pH 6.8 containing 4% SDS, 20% glycerol, 0.02% bromophenol blue and 200 mM DTT as reducing reagent). For antigenic analysis gels were electroblotted on polyvinylidene fluoride (PVDF) membrane. The PVDF membrane was incubated with blocking buffer containing 3% BSA in TBS-Tween 20 (TBS-T 0.1% *v*/*v*) for 2 h followed by incubation with a polyclonal murine antiserum (Abnova) against Hyal-1 diluted 1:2500 with TBS containing 3% BSA for 3 h. The membrane was washed three times using TBS-T for 15 min and subsequently incubated for 2 h in a 1:6500 dilution of the secondary antibody, a HRP linked rabbit anti-mouse IgG. After a renewed washing step with TBS-T the antigen-antibody conjugates were incubated with HRP substrate and visualized using a Chemocam HR16 camera system (Intas, Göttingen, Germany).

## 4. Conclusions

This study is the first report on an active Hyal-1 in *E. coli*, combined with a simple test assay. “Stains-all” assay described here is a sensitive, practical and reliable test. Although recombinant Hyal-1 expression in *E. coli* F470 lacks posttranslational modifications like glycosylation, this whole cell assay yielded a considerable hyaluronidase activity and offers the opportunity of inhibitor screening in microplates. In the present work, we identified three saponins as new Hyal-1 inhibitors with IC_50_ values in the µM range and furthermore, determined the inhibitory effect of selected plant extracts on Hyal-1. Taken together, the whole cell assay reported in this current study will allow a rapid screening of a large number of compounds to obtain a scaffold for the development of further potent inhibitors. Inhibitors of Hyal-1 promise to pave the way for a deeper understanding of how the HA-hyaluronidase system is involved in the biology of cancer and non-cancer related disorders.
